# Artificial intelligence-supported system of surgical anatomy recognition may facilitate the understanding of gastrointestinal surgery for medical students

**DOI:** 10.1007/s00464-025-12205-2

**Published:** 2025-09-13

**Authors:** Shintaro Okumura, Shigeru Tsunoda, Shigeo Hisamori, Shoichi Kitano, Kohei Ueno, Masazumi Sakaguchi, Yu Yoshida, Takashi Sakamoto, Takehito Yamamoto, Ryosuke Okamura, Keiko Kasahara, Masahiro Maeda, Nobuaki Hoshino, Yoshiro Itatani, Koya Hida, Kazutaka Obama

**Affiliations:** Department of Gastrointestinal Surgery, Kyoto University Hospital, 54 Shogoin Kawahara-Cho, Sakyo-Ku, Kyoto, 606-8507 Japan

**Keywords:** Artificial intelligence, Surgical education, Education for medical students

## Abstract

**Background:**

Although the high accuracy of artificial intelligence (AI) for recognizing surgical anatomy has been reported, its effective usage remains unclear. In this study, we investigated the utility of AI in surgical education for medical students.

**Methods:**

Fifth-grade medical students were recruited to investigate the educational utility of EUREKA™. After an introductory lecture, they watched a video of distal gastrectomy with or without the suggestion of the connective tissue and the pancreas by EUREKA™ and then drew dissection lines in still images captured from the video. The distance between the lines drawn by students and the optimal dissection line determined by an expert surgeon was integrated to evaluate how well the students appropriately recognized the dissection line. Students filled out questionnaires after the study. A total of 45 operative video frames from radical gastrectomies performed with three different robotic systems were analyzed. The accuracy of the EUREKA™ recognition of the connective tissue and the pancreas was assessed using Dice and Intersection over Union (IoU) as a measurement tool.

**Results:**

Twelve students participated in the study, and nine students drew dissection lines. All students completed questionnaires. The students could recognize dissection lines more appropriately with the EUREKA™ suggestion, and the deviations between the dissection lines drawn by the students and the optimal dissection lines were significantly reduced. From the questionnaires completed by the students, eight students agreed with the possibility of AI to facilitate their understanding of the operation, and two students agreed with the potential of AI to increase the number of medical students who choose gastrointestinal surgery as their career. There were no differences in the DICE and IoU scores of the connective tissue and the pancreas between the three robotic systems, suggesting the versatility of the EUREKA™ system.

**Conclusion:**

AI may facilitate students’ understanding of surgery.

**Graphical Abstract:**

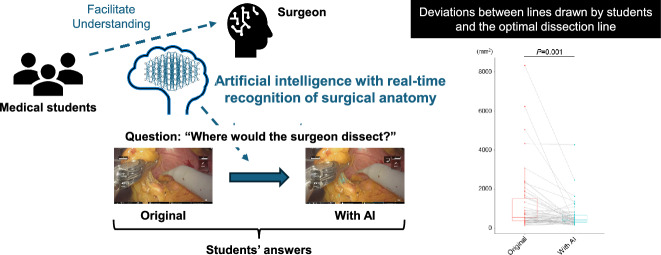

**Supplementary Information:**

The online version contains supplementary material available at 10.1007/s00464-025-12205-2.

In recent years, operative techniques in gastrointestinal surgery have developed along with the evolution of technologies such as optics and robotics [1]. High-resolution endoscopic systems enable surgeons to recognize very fine surgical anatomy and perform sophisticated operations [2, 3]. In robot-assisted surgery, surgeons can manipulate instruments at the intended angle in a stable surgical field [4]. The focus on improving cutting-edge surgical skills has meant that the development of surgical education has been somewhat neglected over the years.

Currently, the clinical application of artificial intelligence (AI) with image recognition has been vigorously investigated [5, 6]. AI with real-time recognition of surgical anatomy is expected to be the next technology to advance gastrointestinal surgery [1]. At Kyoto University Hospital, we recently introduced EUREKA™ (Anaut, Inc.), an AI system with semantic segmentation that was developed through deep learning from numerous videos of gastrointestinal surgeries to recognize surgical anatomies, including the connective tissue, the nerve, and the pancreas [7, 8]. Although EUREKA™ is expected to be applied in surgical education, its effective use has not yet been established. In this study, we investigated the utility of EUREKA™ in surgical education for medical students.

## Materials and methods

### Study design

Fifth-grade medical students who rotated in our department from September 2024 to March 2025 and had not previously observed gastrointestinal surgery were recruited for this study. Written informed consent was obtained from all participants.

In infrapyloric lymph node dissection, it is crucial to correctly identify the connective tissue to be dissected and the pancreas to be preserved. EUREKA™ was reported to recognize both the connective tissue and the pancreas with high accuracy [8, 9]. Thus, we decided to investigate the educational utility of the EUREKA™ suggestion of the connective tissue and the pancreas in infrapyloric lymph node dissection. The study was conducted online using a video of robot-assisted distal gastrectomy with hinotori™ robotic system. Before the beginning of the study, the study participants received a lecture explaining the basic concepts of radical gastrectomy and local anatomy. They were taught how important it is to identify and dissect the loose connective tissue between the fat tissue including the lymph nodes to be harvested and the anatomical structure to be preserved. The participants then watched an original operation video for five minutes and were asked to draw a line that they thought the surgeon would dissect in six still images captured from the video. The process was repeated using the same video, this time with the suggestion of the connective tissue and the pancreas by EUREKA™. Subsequently, the optimal dissection lines determined by an expert surgeon were shown to the participants. The participants were instructed to submit the still images with their dissection lines via email.

### Evaluation of the accuracy of the dissection lines drawn by the students

The distance between the line drawn by the student and the optimal dissection line was integrated from end to end, to quantitatively evaluate how well the student appropriately recognized the dissection line. More specifically, the edges of each line were connected, the area enclosed by the lines was painted red and calculated with Image J, an open-source software for image processing and analysis (https://imagej.net/ij/). A smaller deviation area was considered more accurate.

### Questionnaire after the study

Medical students filled out a web-based questionnaire after finishing the study, answering two questions as follows: “Did AI facilitate your understanding of the operation?” and “Do you think AI would have the potential to increase the number of medical students who choose gastrointestinal surgery as their career?” They were also asked to provide reasons for their answers. Additional information on the questionnaire can be found in the supplementary file.

### Evaluation of the accuracy of the EUREKA™ recognition of surgical anatomy in gastrectomies with three robotic systems

A surgeon sampled 15 frames of key surgical views from three videos of radical gastrectomy for gastric cancer with each of the three robotic systems: Da Vinci Xi, hinotori™, or Hugo™ RAS. A total of 45 frames were analyzed for the recognition of the connective tissue and the pancreas by EUREKA™. Five surgeons with endoscopic surgical skill qualifications from the Japan Society for Endoscopic Surgery manually segmented the corresponding frames from the original videos to obtain the ground truth of each surgical anatomy. The quantitative evaluation of the agreement involved measuring the spatial overlap of pixels between the ground truth and the area recognized by EUREKA™. To evaluate sensitivity and similarity, this assessment employed the Dice and Intersection over Union (IoU). These metrics are calculated as follows:

$$Dice = TP/\left( {TP + 1/2\left( {FP + FN} \right)} \right)$$

$$IoU = TP/\left( {TP + FP + FN} \right)$$

where TP, FN, and FP represent the true-positive, false-negative, and false-positive counts, respectively.

The study protocol for this research project was approved by the Ethics Committee of Kyoto University Hospital (Kyoto University: Study No. R4220) and conforms to the provisions of the Declaration of Helsinki. Informed consent for patients whose operation videos were analyzed was waived due to the retrospective nature of the analysis, and the study information was disclosed on our hospital website, allowing eligible patients to opt out.

### Statistical analysis

Statistical analysis was performed using R (version4.2.2). The Wilcoxon rank-sum test or Wilcoxon signed-rank test was performed to compare variables between the two sample groups. Statistical tests were two-tailed, and statistical significance was set at *P* < 0.05.

## Results

**Fig. 1:**
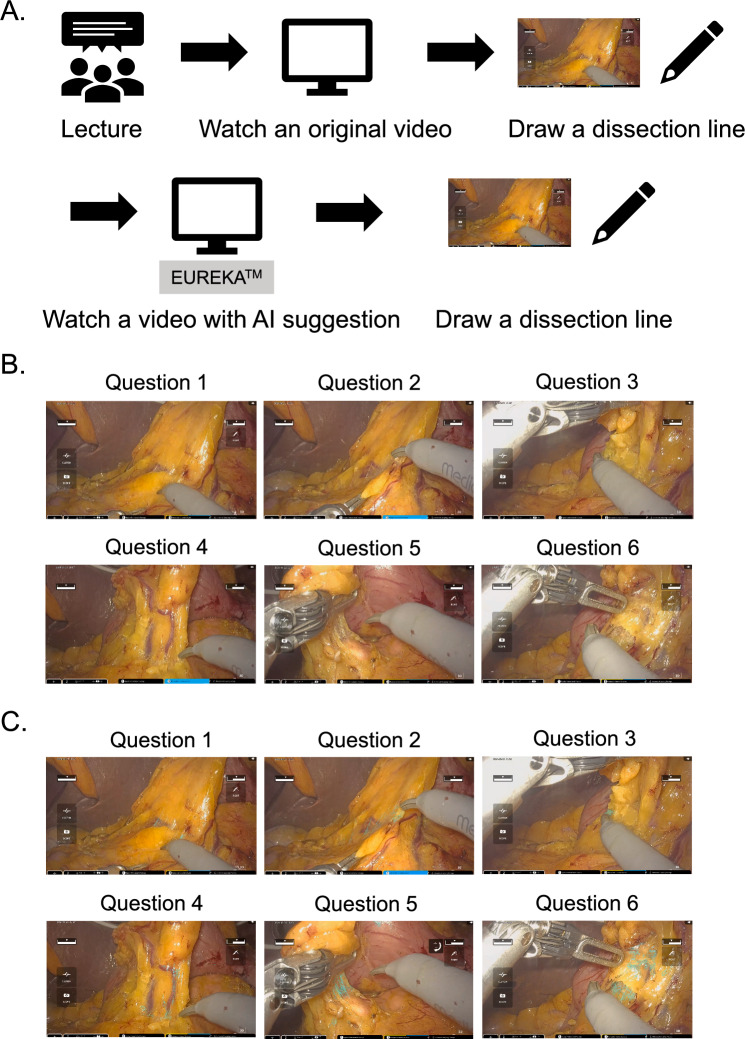
Study design for the utility of AI in surgical education for medical students. **A** The study design. Medical students received a lecture, watched an original operation video, and drew a dissection line in the still images captured from the video. The same process was repeated using the same video with the suggestion of the connective tissue and the pancreas by EUREKA™. **B, C** Six still images were captured from an operation video in the original (**B**) and with the EUREKA™ suggestion (**C**). The connective tissue is colored in blue with EUREKA™ suggestion (**C**)

**Fig. 2:**
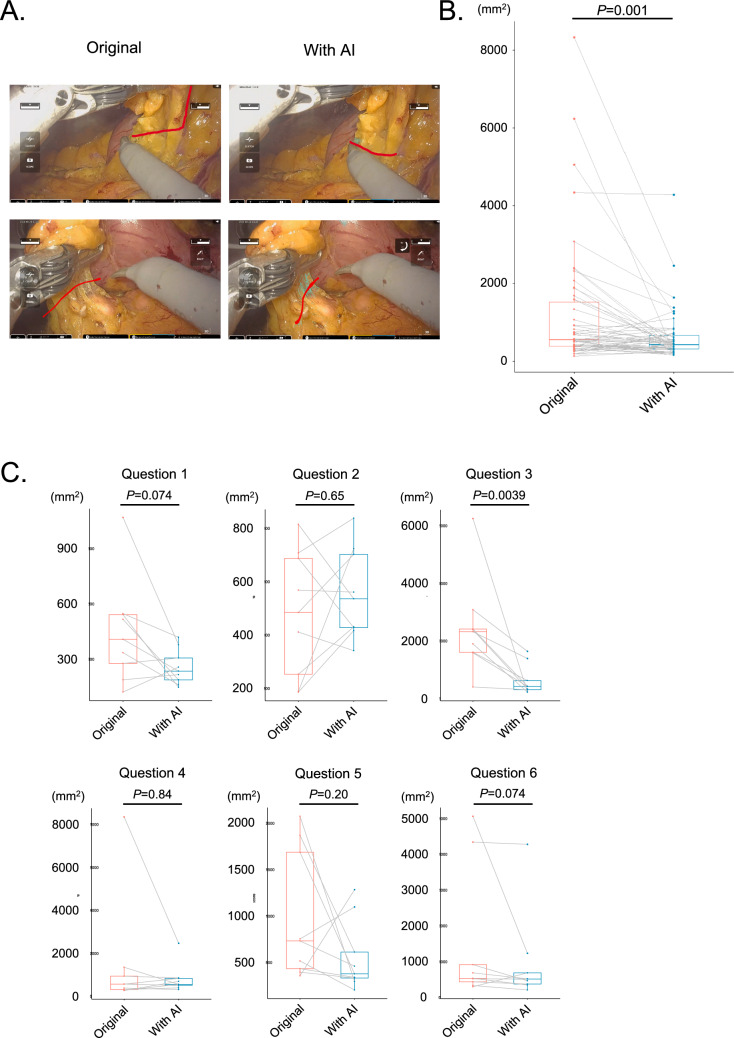
Recognition of dissection lines with or without AI suggestion by the students. The distance between the dissection line drawn by the medical student and the optimal dissection line determined by an expert surgeon was integrated from end to end to evaluate how well the student appropriately recognized the dissection line. **A** Examples of students’ answers for the dissection lines with or without EUREKA™ suggestion. **B** Deviations between the dissection lines drawn by nine students and the optimal dissection lines were compared with and without the EUREKA™ suggestion in all six questions. **C** Deviations between the dissection lines drawn by nine students and the optimal dissection line were compared with and without the EUREKA™ suggestion in each question. The boxes in the graph represent 25th–75th percentiles, lines in the boxes indicate the median; whiskers extend to the maximum and minimum values within 1.5 × the interquartile range; and dots indicate outliers. Statistical significance was determined using a two-tailed Wilcoxon signed-rank test. Statistical significance was set at *P* < 0.05 (**B** and **C**)

Twelve fifth-grade medical students participated in the study. The study participants drew dissection lines in the six still images of the infrapyloric lymph node dissection in robot-assisted distal gastrectomy with or without suggestion of the connective tissue and the pancreas by EUREKA™ (Fig. 1A-C). The study participants were instructed to submit their answers via email, and we obtained answers from nine students. As a whole, deviations between the dissection lines drawn by the nine students and the optimal dissection lines were significantly reduced with the EUREKA™ suggestion of surgical anatomy (*P* = 0.001) (Fig. 2A-B). Thus, it was indicated that students could recognize the dissection lines more appropriately with the EUREKA™ suggestion. When the result of each question was looked at individually, the deviations of Question 3 significantly decreased with the EUREKA™ suggestion (*P* = 0.0039). Those of Question1 (*P* = 0.074) and Question 6 (*P* = 0.074) also decreased with the EUREKA™ suggestion, though not statistically significant. On the contrary, the improvement in deviations was not clearly observed in Questions 2 (*P* = 0.64), Question 4 (*P* = 0.84), and Question 5 (*P* = 0.20).

**Fig. 3:**
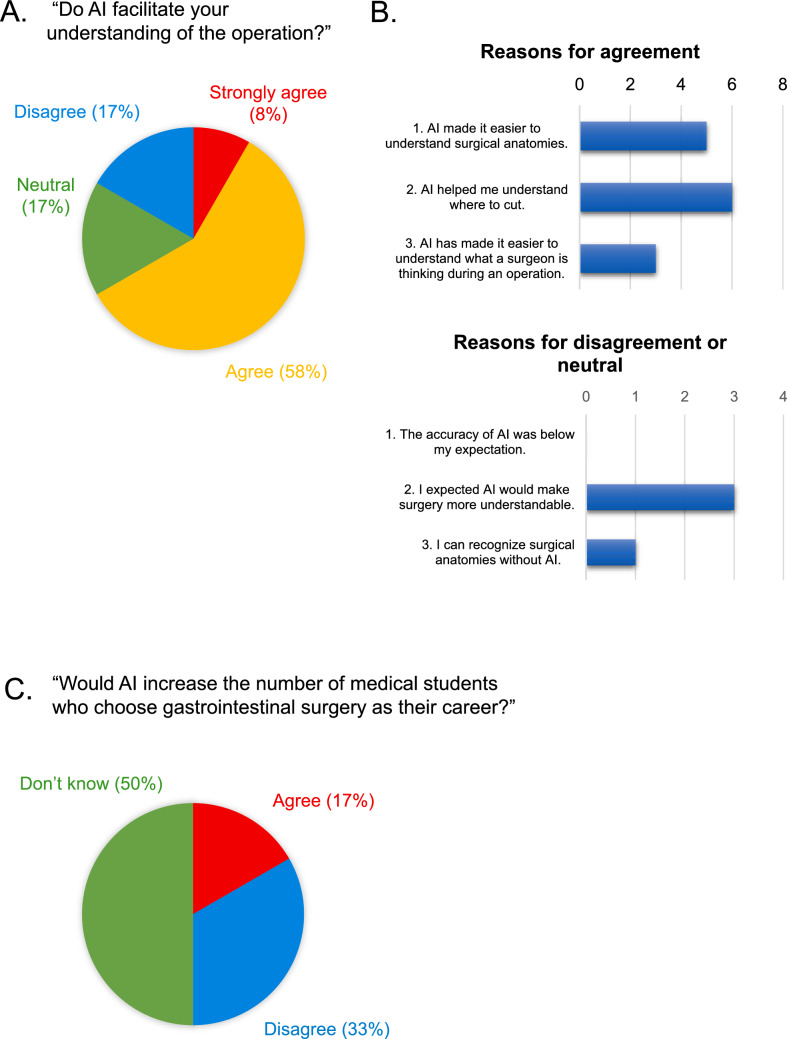
Answers to the questionnaire completed by the students after the study. **A** Distribution of answers to the first question (n = 12). **B** Reasons for their choice of answer. This included multiple reasons for one student. **C **Distribution of the answers to the second question (n = 12)

In addition, we investigated how students’ impressions of gastrointestinal surgery changed with EURKEA™, using a post-study questionnaire. All twelve students completed a web-based questionnaire after the study. Eight students agreed with the possibility of AI to facilitate their understanding of the operation (Fig. 3A), and the most frequent reason was “AI helped me understand where to cut.” (n = 6). Five students selected “AI made it easier to understand surgical anatomies.” In addition, three students selected “AI has made it easier to understand what a surgeon is thinking during an operation” as the reason for the question (Fig. 3B). Although most students gave neutral or negative responses, two students agreed with the potential of AI to increase the number of medical students who choose gastrointestinal surgery as their career (Fig. 3C). In the free form section of the questionnaire, positive reasons were given by some students for their answers as follows: “AI would make medical students more interested in the gastrointestinal surgery”; “AI would lower the psychological hurdle to surgery for medical students.” On the other hand, negative reasons were also given by some students as follows: “there is still a lot to learn from senior doctors rather than AI”; “AI does not improve the harsh working conditions of surgeons or increase the salary commensurate with their workload”.

**Fig. 4:**
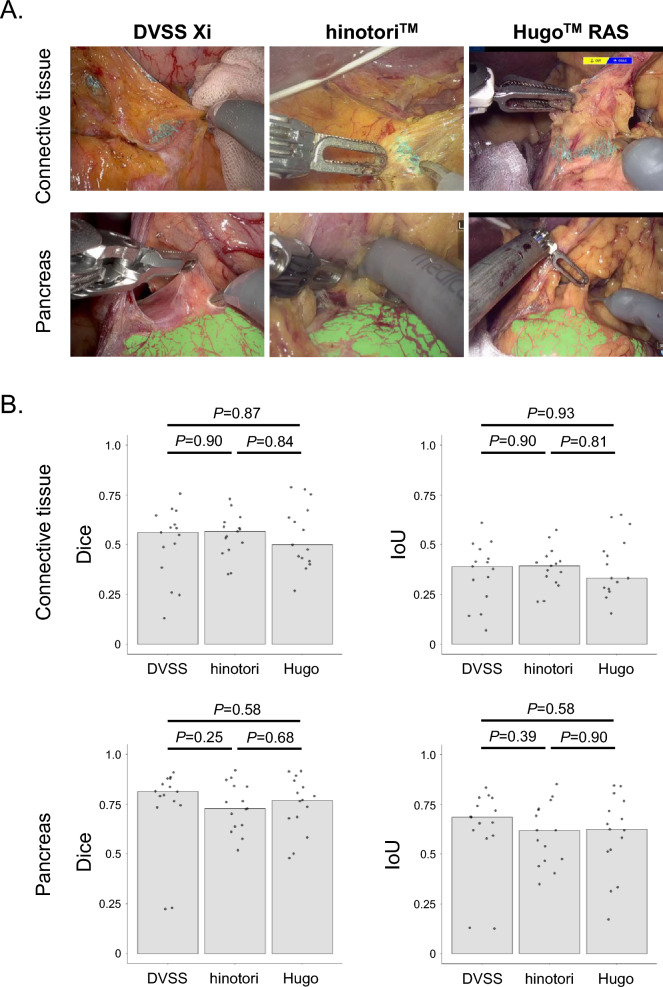
The differences of AI recognition of surgical anatomies between three robotic systems. **A** Representative images of EUREKA™ recognition in the connective tissue (upper panel) and the pancreas (lower panel). Left panel: Da Vinci Xi (DVSS), middle panel: hinotori™ (hinotori), right panel: Hugo™ RAS (Hugo). **B** The accuracies of EUREKA™ recognition of the connective tissue and the pancreas were compared between the three robotic systems with the Dice and Intersection over Union (IoU). Bars indicate the median of the Dice or IoU scores in each group. Statistical significance was determined using a two-tailed Wilcoxon rank-sum test. Statistical significance was set at *P* < 0.05

Finally, we investigated whether EUREKA™ stably recognizes surgical anatomies regardless of the robotic system to study the versatility of EUREKA™. We assessed the accuracy of EUREKA™ for the recognition of the connective tissue and the pancreas in radical gastrectomy with Da Vinci Xi (DVSS), hinotori™ (hinotori), and Hugo™ RAS (Hugo), respectively. There were no differences in the Dice scores of the connective tissue between the three robotic systems (DVSS vs hinotori: *P* = 0.90, DVSS vs Hugo: *P* = 0.87, hinotori vs Hugo: *P* = 0.84) nor in IoU scores (DVSS vs hinotori: *P* = 0.90, DVSS vs Hugo: *P* = 0.93, hinotori vs Hugo: *P* = 0.81). There were also no differences in the Dice scores of the pancreas (DVSS vs hinotori: *P* = 0.25, DVSS vs Hugo: *P* = 0.58, hinotori vs Hugo: *P* = 0.68) nor in IoU scores (DVSS vs hinotori: *P* = 0.39, DVSS vs Hugo: *P* = 0.58, hinotori vs Hugo: *P* = 0.90). (Fig. 4A and B).

## Discussion

Previous studies investigating the utility of AI have focused on the accuracy of the recognition of surgical anatomies but not on the facilitation of understanding of surgery [7–15]. In contrast, this study clearly showed with a quantitative assessment that medical students could recognize the optimal dissection line more appropriately with the EUREKA™ suggestion of the connective tissue and the pancreas.

The scene of infrapyloric lymph node dissection in radical gastrectomy was used to assess surgical understanding of the students. In the infrapyloric area, the gastroduodenal mesenteric tissue and the transverse mesocolon overlap [16]. Fat tissue including the infrapyloric lymph nodes attaches to the surface of the pancreas [17]. These complicated anatomical structures would be difficult for medical students to understand. It may be possible that the educational efficacy of EUREKA™ is conspicuous in procedures with such anatomical difficulty. In fact, the deviations between the dissection lines drawn by the students and the optimal dissection lines were reduced with the EUREKA™ suggestion, especially in the scenes of the right side of the lymph node dissection (Questions 1, 3, and 6). This result corresponds to the fact that the anatomy is more complex on the right side than on the left side of the infrapyloric area.

When measuring the educational usefulness of EUREKA™, we thought that the true answer to whether it is educationally effective might lie in the impressions and opinions of those receiving education. As a result, the answers to the questionnaire of the students also suggested that AI promoted their understanding of the operation and stimulated their interest in gastrointestinal surgery, even though they did not participate in the operation. In recent years, the number of students aspiring to specialize in gastrointestinal surgery has been decreasing. With this in mind, it is significant that among the 12 students, two answered “yes” to the question of whether AI would increase the number of students who choose gastrointestinal surgery as their career. The active use of AI in surgical education for medical students might be effective for their recruitment in gastrointestinal surgery.

Although AI generally involves overfitting, it was revealed that EUREKA™ could stably recognize the connective tissue and the pancreas in gastrectomies performed using three different robotic systems. Therefore, the educational utility of EUREKA™ would be valid regardless of the type of optical and robotic systems used.

This study had some limitations. First, it was conducted with a small number of participants from a single institute. Therefore, a multicenter study with a larger population is needed to clarify the generalizability of the study results. Second, the improvement in recognizing dissecting lines by the students might be due to not only the suggestion of surgical anatomies by EUREKA™, but also the repetition of watching the same videos, although it is difficult to strictly compare the accuracy of students’ recognition of the optimal dissection lines between different operations.

In conclusion, this study proved, for the first time, the possibility of active usage of AI to facilitate students’ understanding of surgery with a quantitative assessment and a questionnaire. It may be possible that using EUREKA™ will enable medical students to autonomously understand surgeries during clinical training, even without detailed real-time explanations from surgeons. Additionally, we believe EUREKA™ would also be useful for surgeons who need to improve their understanding of anatomy or surgical procedures, as they can use instructional videos to review surgeries in advance. We believe that the findings in the current study will promote the utilization of AI in future surgical education and also lead to further multicenter studies with a larger number of participants.

## Supplementary Information

Below is the link to the electronic supplementary material.

Supplementary file1 (DOCX 19 KB)
