# Supplementary material for: Artificial intelligence-supported system of surgical anatomy recognition may facilitate the understanding of gastrointestinal surgery for medical students
Source: Surg Endosc. 2025 Sep 13;39(10):7078–86. doi: 10.1007/s00464-025-12205-2 (PMC12500820; doi:10.1007/s00464-025-12205-2)
Supplement: Supplementary file 1 — Supplementary file1 (DOCX 19 KB) [file 464_2025_12205_MOESM1_ESM.docx]

**Questionnaire**

**Question 1. Did AI facilitate your understanding of the operation?**

1. Agree
2. Strongly agree
3. Neutral
4. Disagree

**Reasons for agreement**

1. AI made it easier to understand surgical anatomies.
2. AI helped me understand where to cut.
3. AI has made it easier to understand what a surgeon is thinking during an operation.
4. Others ( )

**Reasons for disagreement**

1. The accuracy of AI was below my expectation.
2. I expected AI would make surgery more understandable.
3. I can recognize surgical anatomies without AI.
4. Others ( )

**Question 2. Do you think AI would have the potential to increase the number of medical students who choose gastrointestinal surgery as their career?**

1. Agree
2. Don’t know
3. Disagree

**Reasons for the answer (Free form)**

**( )**
